# Celebrating the past, concentrating on the future: the next decade for AR&T

**DOI:** 10.1186/s13075-015-0812-1

**Published:** 2015-10-15

**Authors:** Christopher D. Buckley, Harris Perlman

**Affiliations:** Rheumatology Research Group, Institute for Translational Inflammation Research, College of Medical and Dental Sciences, University of Birmingham Research Laboratories, Queen Elizabeth Hospital Birmingham, Birmingham, B15 2WD UK; Department of Medicine, Division of Rheumatology, Feinberg School of Medicine, Northwestern University, 240 E. Huron Street, McGaw M338, Chicago, IL 60611 USA

Rheumatology is at a very exciting stage in its long and illustrious history. New biological therapies have revolutionized the treatment of immune-mediated inflammatory diseases. The genetic, epigenetic and environmental mechanisms that drive musculoskeletal diseases are giving up their secrets, and patients are much more involved in the decisions that matter to them… “nothing about us without us”. These rapid advances in musculoskeletal medicine suggest that finally Cinderella has come to the ball!

Musculoskeletal medicine is a broad field, which encompasses many clinical and scientific disciplines that are already served by excellent journals. So what is unique about *Arthritis Research & Therapy* (AR&T) and whom is the journal aimed at?

In its first decade, AR&T under the guidance of its founding Editors has established itself as a leading journal for the community of researchers and clinicians interested in the science of musculoskeletal medicine. It is open access, links basic science with clinical advances and enjoys independence and a freedom to be innovative; for example, it was the first open-access journal in the field. We believe that this is key for our continued growth.

What will be our editorial focus as we work with our illustrious editorial team to steer the journal as it enters its second decade? Our goal is to ensure that the journal serves and is served by a loyal and engaged scientific and clinical community. That is why we have followed our founders’ policy of having two Editors-in-Chief: one a clinician, the other a scientist. We also believe that, more than ever before, the best developments and discoveries occur through collaborations across various academic centres worldwide. This is why our two Editors are from different continents and our Section Editors are from all over the world. We will continue to seek out genuine translational science that has delivered such stunning advances and at the same time we will adapt to changes in the scientific landscape. To achieve this, we have set ourselves the following objectives.

Firstly, we will continue to expand the remit of the journal to include other rheumatic diseases such as osteoarthritis, crystal arthritis, lupus and the connective tissue diseases. Although AR&T has traditionally focused on translational research, it has not published many pivotal clinical trials. Therefore, we will try to publish the best clinical trials and outcome studies, including those that report on health economic evaluation.

Secondly, we aim to continue to publish research of the highest quality related to basic biological processes. We will accomplish this by attracting manuscripts that report the use of cutting-edge technologies that link clinical and radiologic phenotypes with genomic, transcriptomic, epigenomic and proteomic measurements in clinical samples from patients with musculoskeletal disease, thereby generating new insights and hypotheses about disease pathogenesis.

Thirdly, we will reinvigorate our reviewer base by promoting the role of junior faculty and, in particular, postdoctoral fellow researchers. These young investigators represent the future of our specialty and need our encouragement and support as they work to generate insights and therapies for the future. We would like these young members of the journal to become the new backbone of our review process. We will also highlight the research of junior fellows by using podcasts. This junior generation are our future and they deserve attention in promoting their outstanding work. They are very competent and comfortable with new technologies yet often feel undervalued and under-represented.

Fourthly, we will work with specialty societies to highlight novel ongoing research at their meetings in an aim to expand the research scope and reportage of the journal.

Finally, we will focus on editorials that highlight cutting-edge areas of rheumatic disease that are published in our journal as well as others.

So how will we achieve these goals? We have established a new editorial structure with an international selection of outstanding and expert investigators. These new Section Editors will oversee six general research themes: David Pisetsky (USA) – Immunology and Pathology; Maya Buch (UK) – Pharmacology and Therapeutics; Laure Gossec (France) – Epidemiology and Clinical Trials; Edward Schwarz (USA) – Outcomes and Imaging; Kazuhiko Yamamoto (Japan) – Genetics and Epigenetics; and Thomas Pap (Germany) – Bone and Cartilage Biology.

We have moved over to an easy-to-use online editorial management system that will improve our governance and editorial consistency and enhance the turnaround rate for manuscripts. It will also allow us to monitor the review process and tell us whether we are accomplishing what we said we would. We have refreshed and will continue to revise our pool of Associate Editors and Board Members so that we have more involvement from junior faculty. We are also fully aware that reviewer fatigue is a problem throughout academia. Therefore, our Associate Editors and Board Members will commit to reviewing at least six manuscripts a year. They will also act as our advocates for the journal to the scientific, clinical and patient populations that we serve.

Our ultimate aim is to be not only a trusted, internationally respected rheumatic disease journal but one that expands the boundaries of musculoskeletal disease research and identifies with an empowered scientific and clinical community. Thus, although impact factors do matter, we are not obsessed by them. We believe that by engaging with our research community we will attract the best research that will ultimately lead to an increased impact factor and enhance our international recognition. As the new Editors we invite you to join us in this new mission for AR&T.

